# Gaps between research and public health priorities in low income countries: evidence from a systematic literature review focused on Cambodia

**DOI:** 10.1186/s13012-015-0217-1

**Published:** 2015-03-11

**Authors:** Sophie Goyet, Socheat Touch, Por Ir, Sovannchhorvin SamAn, Thomas Fassier, Roger Frutos, Arnaud Tarantola, Hubert Barennes

**Affiliations:** Epidemiology and Public Health Unit, Institut Pasteur, Phnom Penh, Cambodia; School of Public Health, National Institute of Public Health, Phnom Penh, Cambodia; Health System Research and Policy Support Unit, National Institute of Public Health, Phnom Penh, Cambodia; University of Health Sciences of Cambodia, Phnom Penh, Cambodia; UM2, CPBS, UMR 5236, CNRS-UM1-UM2, 1919 route de Mende, 34293 Montpellier, Cedex 5, France; Intertryp, UMR 17, IRD-Cirad, Campus International de Baillarguet, 34398 Montpellier, Cedex 5, France; INSERM, ISPED, Centre INSERM U897-Epidemiologie-Biostatistique, Université de Bordeaux, F-33000 Bordeaux, France; Agence Nationale de Recherche sur le VIH et Hépatite, ANRS, Phnom Penh, Cambodia

**Keywords:** Health, Health system strengthening, Public health, Research, Knowledge translation, Developing countries, LMICs, Non-communicable disease, Asia, Cambodia

## Abstract

**Background:**

Evidence-based public health requires that research provides policymakers with reliable and accessible information reflecting the disease threats. We described the scientific production of research in Cambodia and assessed to what extent it provides appropriate insights and implications for practice to guide health policymakers and managers and knowledge relevant for translation.

**Methods:**

We conducted a systematic review of scientific articles published on biomedical research in Cambodia. Regression analysis assessed the trends over time and factors associated with actionable messages in the articles’ abstracts.

**Results:**

From 2000 to 2012, 628 articles were published in 237 journals with a significant increase over time (from 0.6/million population to 5.9/million population, slope coefficient 7.6, 95% CI 6.5–8.7, *p* < 0.001). Most publications on diseases addressed communicable diseases (*n* = 410, 65.3%). Non-communicable diseases (NCD) were under-addressed (7.7% of all publications) considering their burden (34.5% of the disease burden). Of all articles, 67.8% reported descriptive studies and 4.3% reported studies with a high level of evidence; 27.4% of studies were led by an institution based in Cambodia. Factors associated with an actionable message (*n* = 73, 26.6%) were maternal health (OR 3.08, 95% CI 1.55–6.13, *p* = 0.001), the first author’s institution being Cambodian (OR 1.78, 95% CI 1.06–2.98, *p* = 0.02) and a free access to full article (OR 3.07, 95% CI 1.08–8.70, *p* = 0.03). Of all articles, 87% (*n* = 546) were accessible in full text from Cambodia.

**Conclusions:**

Scientific publications do not fully match with health priorities. Gaps remain regarding NCD, implementation studies, and health system research. A health research agenda would help align research with health priorities. We recommend 1) that the health authorities create an online repository of research findings with abstracts in the local language; 2) that academics emphasize the importance of research in their university teaching; and 3) that the researcher teams involve local researchers and that they systematically provide a translation of their abstracts upon submission to a journal. We conclude that building the bridge between research and public health requires a willful, comprehensive strategy rather than relying solely only publications.

**Electronic supplementary material:**

The online version of this article (doi:10.1186/s13012-015-0217-1) contains supplementary material, which is available to authorized users.

## Introduction

The gap between research and public health priorities is a challenge for researchers and policymakers worldwide [1-3]. This gap is especially wide in developing countries where evidence-based information is scarce [4,5]. Although the number of new scientific articles published worldwide each year is considerable, a limited proportion of these articles report studies from low- and middle-income countries (LMICs), where most preventable deaths occur [6]. In 2000, the total share of publications coming from LMICs was estimated at 1.7%. This shows an impressive imbalance between developed and developing countries, although recent trends suggest that scientific production is rising in countries in transition such as China, India, and Brazil [7-9].

Bridging the gap between research and policymaking requires knowledge translation (KT) strategies. KT is defined as “the use of knowledge in practice and decision-making by the public, patients, healthcare professionals, managers, and policymakers” [10]. KT is a non-linear process based on iterative steps [11], the first essential step being the generation, synthesis, and sharing of scientific knowledge produced by research. Indeed, policymakers need to access studies with high level of evidence, using data reflecting the current burden of disease of anticipated threats, in journals accessible easily and in a timely manner to help base their public health decisions on evidence. Policymakers also need clear statements of research findings and implications for practice [12,13]. The World Health Organization (WHO) recommends the use of policy briefs and actionable messages to prompt effective communication of research findings to the most appropriate target audiences [14,15]. Actionable messages are action-oriented recommendations specifying the actor and/or the target. To our knowledge, there is no standardized tool to assess the presence of actionable messages in publications on evidence-informed policymaking.

Cambodia, a Southeast Asian LIC, is currently experiencing the multiple and complex demographic and epidemiologic changes that are associated with countries in transition [16]. Cambodia’s demographic transition began in the mid-2000s with a rapid decrease in the birth rate and increases in population aging and rural-to-urban migration. The decade 2000–2010 also witnessed an epidemiological transition characterized by a shift from infectious to non-communicable diseases (NCD) and a neglected epidemic of road injuries, both of which have challenged the national health system [17,18]. In this rapidly evolving but fragile context, addressing the gap between research and policymaking is crucial when seeking to use an evidence-based approach to define the public health priority strategies.

In Cambodia, health research remains limited to a few national institutions (National Institute of Public Health (NIPH), University of Health Sciences (UHS), Pasteur Institute in Cambodia…) and some other actors (international researchers collaborating with hospitals, non-governmental organizations). There is neither a systematic description of the health research output in Cambodia nor an assessment of its potential for guiding public health policymaking.

The aim of this study was to perform a systematic review of the literature about health research in Cambodia published in peer-reviewed scientific journals from 2000 to 2012. This careful, collaborative analysis of publications by national and international teams was conducted to serve as a baseline to measure progress in developing research capacity and harmonizing research with public heath priorities. The review therefore had dual objectives of (1) describing the articles published health-related issues in Cambodia and (2) assessing their potential for guiding health policymaking.

## Method

### Searching, retrieving, and selecting the literature

In October 2012, we searched the Medline database via the PubMed search engine following a search strategy using the keywords “Cambodia” or “Cambodian” (Additional file 1). The timeframe was from 2000 up to and including the date of the search. Since most Cambodian physicians were, until recently, taught in French at university, we complemented this search by performing a French-language search of the Directory of Open Access Journals (DOAJ) and scientific journals on tropical medicine in January 2013.

Eligible articles were 1) related to research conducted in Cambodia and 2) related to human health or the health system in Cambodia. We excluded articles solely presenting research findings on pure botany, chemistry, physics, or zoology.

### Data extraction and management

Article identifiers were downloaded from the sources (year of publication, journal, name of authors, name and country of first author’s institution). All eligible abstracts were downloaded. The information on articles’ topics, type of research, type of data, level of evidence and content, were independently extracted from the abstracts by two researchers using a standardized form which was pilot-tested on 200 abstracts and refined accordingly. Discrepancies between the two researchers were systematically explored, and a consensual decision was reached in all cases after team discussions. Data were entered into an in-house EpiData database format.

### Definitions and classifications

To describe the articles, the following definitions and methods were used:

The *main topic* of articles was classified as “diseases/conditions” or “health system” or both. *Subtopics* of articles related to disease/condition issues were classified according to the Global Burden of Disease Study [19]. Health system subtopics were organized according to the WHO building block framework: health service provision, human resources, pharmacy, governance, financing and equity, and information system [20].

*Type of research* was classified into three groups [21]. *Descriptive research* included studies on incidence or prevalence, risk factors, knowledge attitude practices, healthcare practices, and health policies. *Implementation research* included studies assessing the efficacy, the efficiency, the replication, and the dissemination of interventions. “*Fundamental research*” focused on laboratory research, studies assessing measurement instruments and studies exploring a topic from the sole physiological perspective. We also classified modeling studies (e.g., work focusing on the impact of preventive actions against a disease onto the health system) as fundamental research.

Data types were classified as (1) *original research* when containing original data; (2) *derivative* if it was a secondary analysis of original data; and (3) *non-original* when concerning reviews and viewpoints.

*Origin* of research was defined as “Cambodian” or “foreign” according to the country of the first author’s affiliated institution, as indicated by the authors.

The *impact* of publications was assessed using the latest available journal impact factor (2012 IF) [5,6] and the Article Influence® (AI) score. The AI is a measure of per-article citations weighted by influence in a given journal. A score greater than 1.00 indicates that the article is above-average influence in the journal. The 2010 IF and the 2012 AI were extracted from the SCImago Journal & Country Rank internet portal [22].

To assess the publications’ potential to inform the policymaking, the following definitions and methods were used:

*Level of evidence* of studies has been classified, based on their study design, sorted by their susceptibility of bias according to the Scottish Intercollegiate Guidelines Network (SIGN) grading system [23]. Systematic reviews and meta-analysis of randomized controlled trials (RCT) were awarded the highest class, followed by non-randomized intervention studies and observational and non-experimental studies. The lowest class was given to expert opinion.

Following the approach adopted in a recent Indian study [24], we assessed *the adequacy between publications and the burden of diseases* by comparing the proportion of articles addressing each disease with the country-wide burden as estimated by the Global Burden of Disease Study (GBDS) [19]. Because more recent GBDS data was not available for Cambodia, the 2004 Study was used.

Abstracts were screened in search of *recommendations* to policymakers, healthcare providers, researchers, and patients. We then classified those messages as 1) “*Actionable messages*” if they were action-oriented and specified the actor and/or the target; and 2) “*Implications*” if no actionable message was clearly stated but a need for action was identified.

*Accessibility* was systematically checked via the PubMed Central repository, via the Health Inter-Network Access to Research Initiative (HINARI) website (a WHO-sponsored website providing free access to some articles for LMIC) and via the journal websites, or the Google Scholar search engine.

### Analysis

A flow chart summarizing the publication selection process was done according to the Preferred Reporting Items for Systematic Reviews and Meta-Analyses (PRISMA) recommendations [25]. Publication characteristics were described with medians, interquartile ranges (IQR), or numbers and percentages. Raw numbers of publications were normalized by the population of Cambodia in 2000 and 2010. Trends in publications were assessed using linear regression analyses. A positive and statistically significant slope coefficient showed an increase over time. Regression analyses only covered the period 2000–2011 as the study period did not cover 2012 in full. A univariate analysis of factors associated with the presence of an actionable and targeted message in the abstract was performed using logistic regression and reported odd ratios (OR) and their confidence intervals (95% CI). Significance of statistical tests was assessed at *P <* 0.05. Data were analyzed using Stata 13.1 software (College Station, Texas).

### Ethics

No approval by a human-subject review board was required for this desk review of published articles.

## Results

**Figure 1:**
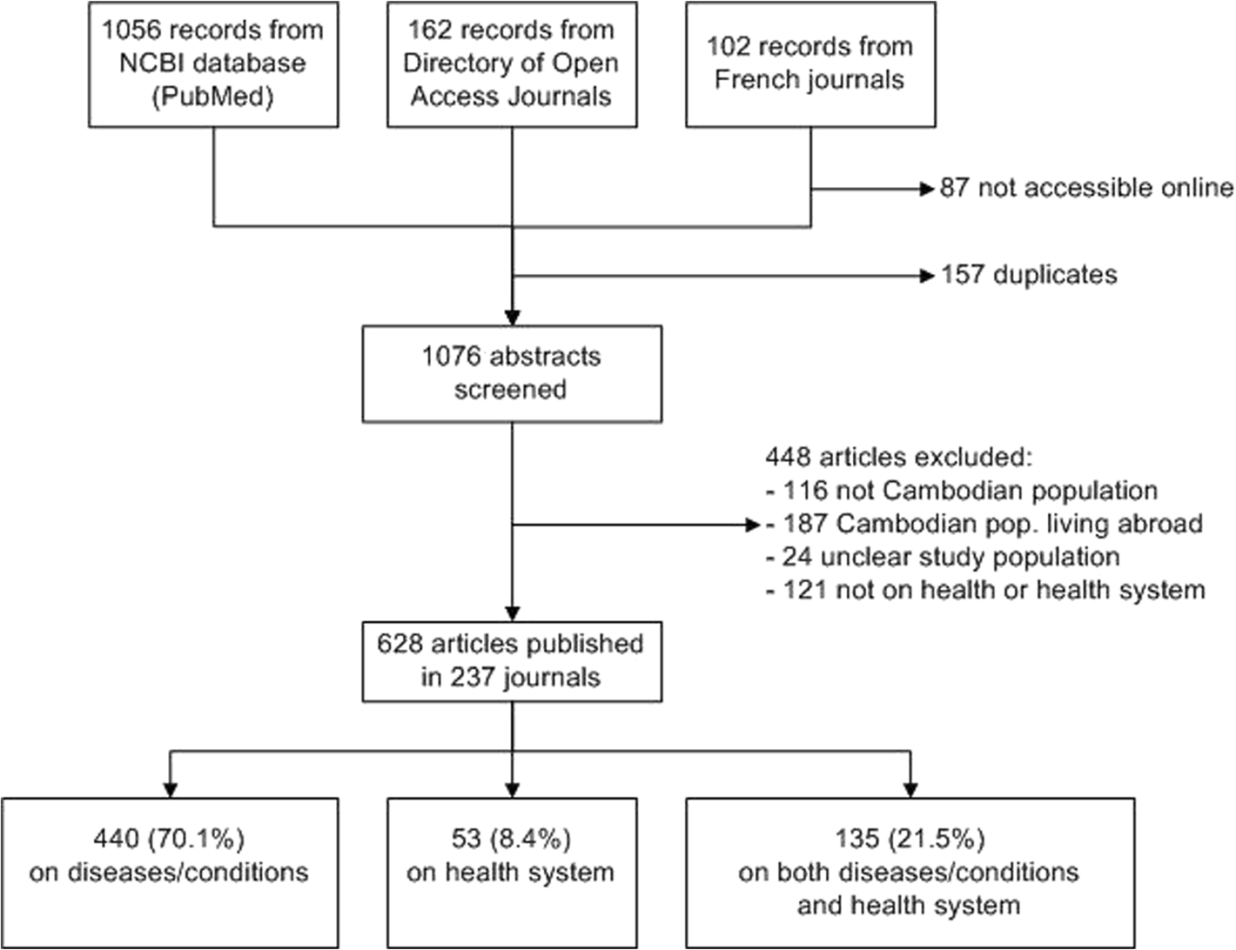
**PRISMA flow chart.**

From January 2000 to October 2012, 628 scientific articles were published in 237 journals referenced in PubMed, the DOAJ, or French journals (Figure 1). Of 628 publications, 440 (70.1%) related to diseases/conditions, 53 (8.4%) to the health system, and 135 (21.5%) to both.

### Description

**Figure 2:**
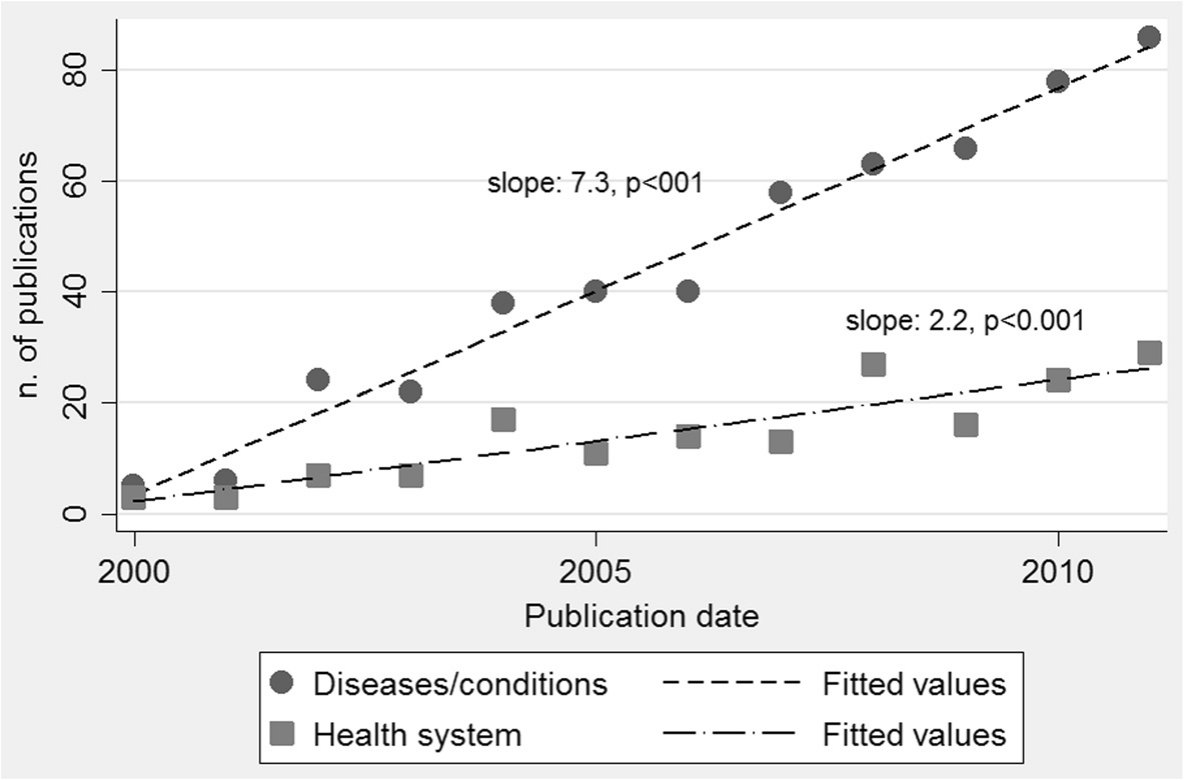
**Trends in health research production in Cambodia, 2000–2011.** Simple linear regression analysis was performed to estimate the slope coefficients. The slope coefficients, estimated by simple linear regression analysis, were estimated at 7.2 (95% CI 6.5–8.7, *p* < 0.001) for publications on health and at 2.2 (95% CI 1.4–3.0, *p* < 0.001) for publications on the health system research. Population figures: 12.2 million population in 2000 (http://data.worldbank.org/country/cambodia, projections estimates from 1998 national census) and 14.4 million population in 2010 (http://databank.worldbank.org/data/views/reports/tableview.aspx).

Both the crude and normalized number of publications significantly increased between 2000 and 2011 (Table 1). From 2000 to 2011, the total number of publications increased from 0.6 to 5.9 per million populations, with a stronger increase for publications on diseases and health conditions (Figure 2).

**Table 1:** **Research by topics and by population, Cambodia, PUBLICAM study**

**Topics**	**2000–2012**		**In 2000** ^**a**^		**In 2010** ^**b**^		**Trends 2000–2011** ^**c**^	
	***n***	**%**	***n***	***n*** **/ million population**	***n***	***n*** **/ million population**	**Slope coefficient (95% CI)**	***p*** **values**
All articles	628	100.0	7	0.6	85	5.9	7.6 (6.5–8.7)	<0.001
Diseases/conditions topics only	440	70.1	4	0.3	61	4.2	-	-
Health system topics only	53	8.4	2	0.2	7	0.5	-	-
Both diseases/conditions and health system topics	135	21.5	1	0.1	17	1.2	-	-
Diseases/conditions topics^d^	575	91.6	5	0.4	78	5.4	7.2 (6.5**–**8.0)	<0.001
Communicable diseases	410	65.4	4	0.3	55	3.8	5.4 (4.5–6.3)	<0.001
Non-communicable diseases	44	7.0	0	0.0	6	0.4	0.6 (0.3–0.8)	<0.001
Maternal and reproductive health	49	7.8	0	0.0	7	0.5	-	-
Perinatal conditions	7	1.1	0	0.0	0	0.0	-	-
Nutrition	39	6.2	0	0.0	4	0.3	-	-
Injuries and traumatisms	45	7.0	1	0.1	3	0.2	0.3 (−0.0–0.6)	0.08
Other health problems	14	2.2	0	0.0	3	0.2	-	-
Unspecified health problems	13	2.1	0	0.0	1	0.1	-	-
Indicators, determinants, or needs	2	0.3	0	0.0	0	0.0	-	-
Health system topics^d^	187	29.8	3	0.2	24	1.7	2.2 (1.4–3.0)	<0.001
Health services delivery	85	13.5	1	0.1	12	0.8	1.2 (0.6–1.8)	<0.001
Governance and leadership	63	10.0	2	0.2	9	0.6	0.5 (0.2–0.9)	0.01
Finances and equity	41	6.5	0	0.0	7	0.5	0.6 (0.2–0.9)	0.003
Human resources	33	5.3	1	0.1	4	0.3	0.4 (0.2–0.6)	0.002
Pharmacy and technologies	29	4.6	0	0.0	2	0.1	0.4 (0.0–0.8)	0.05
Information system	16	2.5	0	0.0	2	0.1	0.3 (0.1–0.5)	0.01

^a^12.2 million population in 2000, source http://data.worldbank.org/country/cambodia, projections estimates from 1998 national census.

^b^14.4 million population in 2010, source http://databank.worldbank.org/data/views/reports/tableview.aspx.

^c^We did not include the year 2012 in this analysis as the study period did not cover the full year.

^d^Articles can relate to multiple subtopics.

### Topics covered

Most publications on diseases/conditions addressed communicable diseases (CD) (*n* = 410, 65.3% of all 628 publications) (Table 1). The five most often addressed CD were HIV/AIDS (*n* = 138, 24.0% of articles on diseases and conditions), malaria (*n* = 88, 15.3%), tuberculosis (*n* = 43, 7.5%), intestinal nematode infections (*n* = 41, 7.1%), and influenza (*n* = 32, 5.6% publications) (Additional file 2: Table S1). Articles on NCD (*n* = 44, 7.0%) mainly reported studies on congenital diseases (*n* = 13, 2.3%), malignant neoplasms (*n* = 11, 1.9%), and diabetes (*n* = 5, 0.9%). Research on reproductive health mostly addressed family planning (*n* = 22, 3.8%).

**Figure 3:**
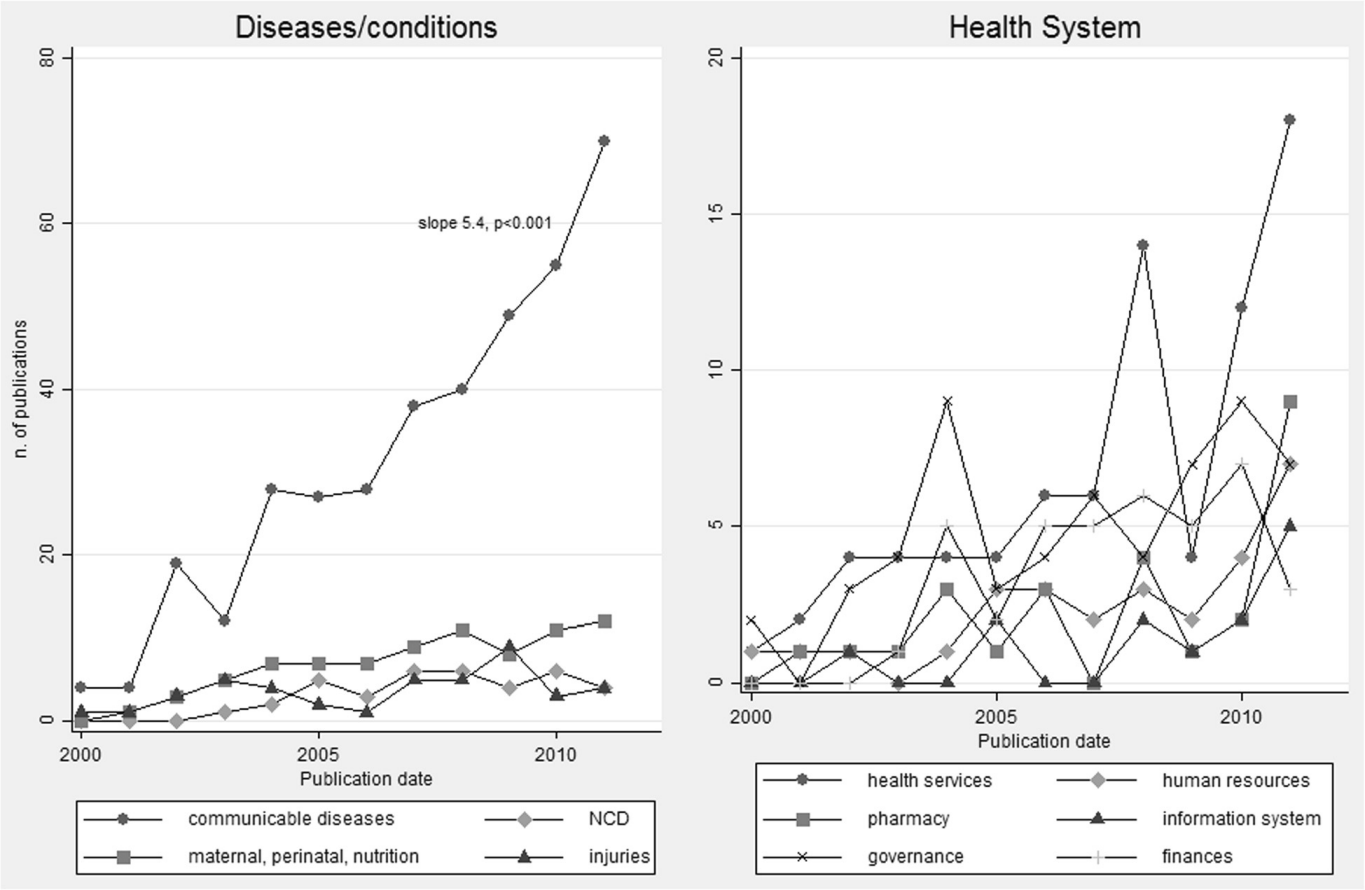
**Trends in publications by subtopics, 2000–2011.**

The analysis of trends over time showed that CD contributed most to the significant increase in the number of publications on diseases/conditions (Figure 3).

The majority of health system articles (*n* = 158, 29.9%) covered the topics of health service delivery (*n* = 85, 13.5% of all 628 articles) and governance (*n* = 63, 10.0%) (Table 1).

### Type of research

Most publications reported descriptive studies. Studies of this type accounted for *n* = 282 (64.1%) of the articles that addressed diseases/conditions and for *n* = 144 (76.6%) of articles on the health system (*p* = 0.002) (Table 2). The proportion of articles reporting implementation studies was higher in articles addressing health system research articles than articles focused solely on diseases/conditions only (*n* = 40/188, 21.3% vs *n* = 63/440, 14.3%, *p* = 0.03). Fundamental studies accounted for 15.8% (*n* = 99/628) of all articles.

**Table 2:** **Study design, data source, and level of evidence of 628 publications on diseases/conditions and health system research in Cambodia, PUBLICAM study, 2000 to 2012**

	**All**	**On diseases/conditions only**	**On health system only**	**On both**
	***n*** **(%)**	***n*** **(%)**	***n*** **(%)**	***n*** **(%)**
All	628 (100.0)	440 (100.0)	53 (100.0)	135 (100.0)
Study design				
Descriptive research	426 (67.8)	282 (64.1)	44 (83.0)	100 (74.1)
Implementation research	103 (16.4)	63 (14.3)	9 (17.0)	31 (23.0)
Fundamental research	99 (15.8)	95 (21.6)	0	4 (3.0)
Data source				
Original	490 (78.0)	370 (84.1)	37 (69.8)	83 (61.5)
Derivative	49 (7.8)	23 (5.2)	4 (7.5)	22 (16.3)
Non-original	52 (8.3)	25 (5.7)	9 (17.0)	18 (13.3)
Unknown/non-appropriate^a^	39 (6.2)	22 (5.0)	3 (3.7)	12 (8.9)
Level of evidence				
Systematic reviews and RCT	27 (4.3)	21 (4.8)	1 (1.9)	5 (3.7)
Non-randomized interventions	11 (1.8)	6 (1.4)	1 (1.9)	4 (3.0)
Observational and non-systematic reviews	550 (87.6)	394 (89.6)	47 (88.7)	109 (80.7)
Expert opinions	13 (2.1)	7 (1.6)	2 (3.8)	4 (3.0)
Others^b^	27 (4.3)	12 (2.7)	2 (3.8)	13 (9.6)

^a^Data source was classified as non-appropriate for articles presenting study protocols.

^b^Other types of publications include study protocols and unknown type (when the abstract contained only few words).

Most publications based their analyses on original data (*n* = 490, 78.0%) while some were reviews of existing data (*n* = 52, 8.3%) or data computed from previously reported information (e.g., the Cambodian Demographic Health Surveys for instance) (*n* = 49, 7.8%) (Table 2).

### Origin of research

**Figure 4:**
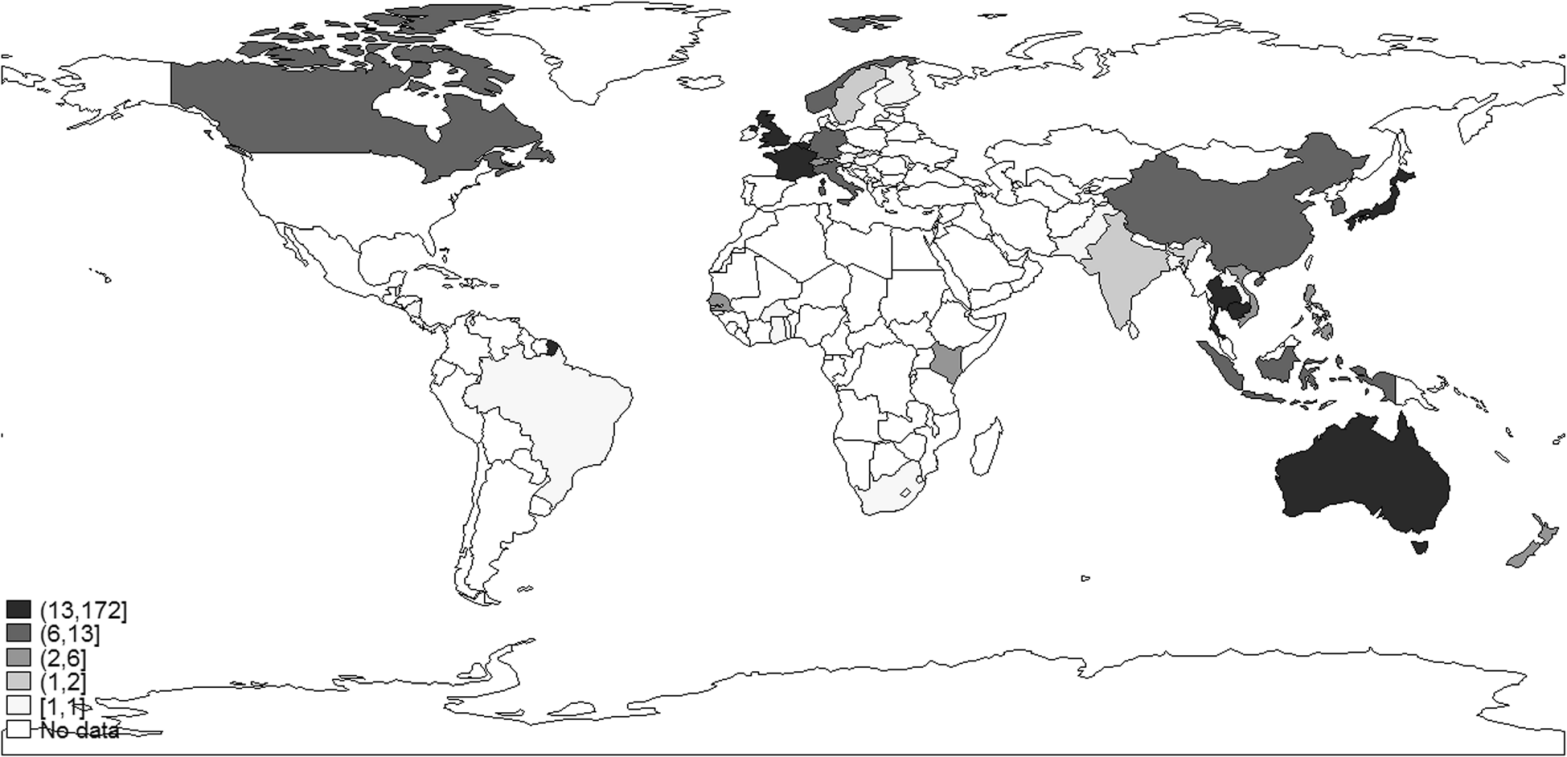
**Country of corresponding authors’ institutions, 2000–2012.**

In 173 articles (27.5%), the first author was affiliated to an institution based in Cambodia. The next top 5 countries leading research were the USA (*n* = 107, 17.0%), Japan (*n* = 49, 7.8%), Thailand (*n* = 45, 7.2%), France (*n* = 44, 7.0%), and Australia (*n* = 36, 5.7%) (Figure 4). The most productive institutions based in Cambodia were the national public institutions and hospitals (*n* = 69, 40.1%) and the Pasteur Institute in Cambodia (*n* = 51/172, 29.5%). The proportion of publications led by a Cambodian institution tended to increase over time (slope 1.9, 95% CI 0.9–2.7, *p* = 0.001).

### Impact

The journal impact factor (IF) was documented for 477 (75.9%) articles. The median IF was 2.82 (IQR 2.11–4.22). Twenty-three (3.7%) articles were in the top 5% journals (IF >10.191). These included the Lancet journals (*n* = 14/23), the New England Journal of Medicine (*n* = 4/23), JAMA (*n* = 2/23), PLoS Medicine (*n* = 1/23), Blood (*n* = 1/23) and Science (*n* = 1/23). Just less than half of the articles published in these journals addressed only diseases/conditions (*n* = 12/23).

The AI score was known for 579 (92.2%) articles. The AI was higher than 1.00 for 294 (46.8%) articles. Of these, 70.8% (*n* = 208) related solely to diseases/conditions and 93.2% (*n* = 274/294) were freely accessible in full text.

### Potential to inform health policymaking

#### Level of evidence

The majority (*n* = 550, 87.6%) of publications reported a medium level of evidence (observational studies or non-systematic reviews). Publications presenting high levels of evidence (systematic reviews and randomized controlled trials) represented 4.3% (*n* = 27) of all articles and addressed either diseases/conditions or health system issues (Table 2).

#### Adequacy between publications and burden of diseases

**Figure 5:**
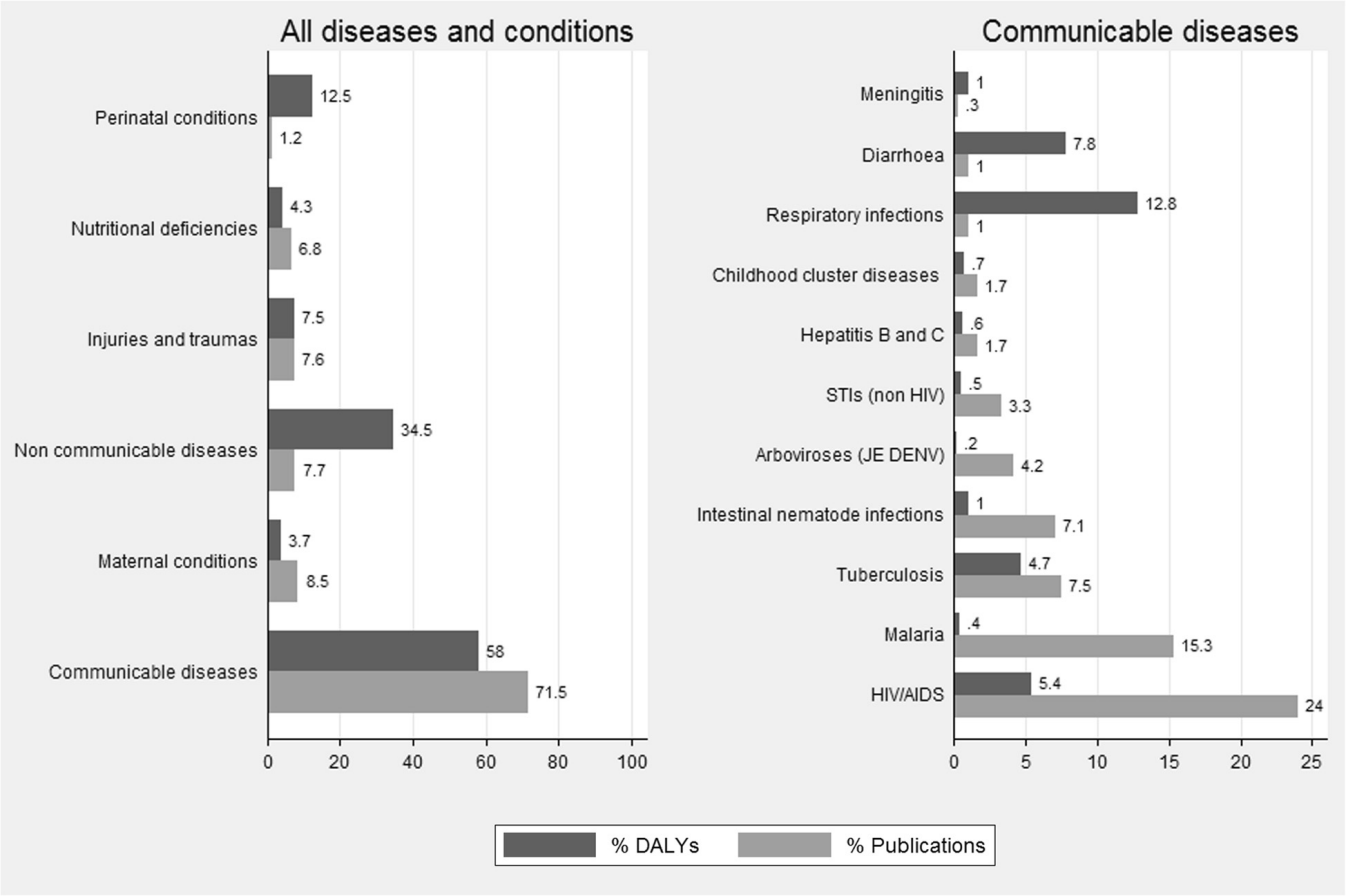
**Proportion of publications by topics compared to the contribution to the 2004 burden of diseases in DALYs, 2000–2012.**

Comparisons with the 2004 burden of disease (in disability adjusted life years (DALYs)) showed some discrepancies between the contribution of some diseases and their representation in the published literature (Figure 5, Additional file 3). While NCD and perinatal conditions contribute to 34.5% and 12.5% of DALYs per 100,000 populations respectively, they were the subject of only 7.7% and 1.2% of all publications on diseases/conditions. Communicable diseases, maternal and reproductive health, and nutritional deficiencies were more frequently studied in publications than their estimated burden on health. The highest discrepancies between publications and the 2004 burden of disease were found among CD, with HIV and malaria contributing to 5.4% and 0.4% of the 2004 burden respectively, while they were addressed by 24.0% and 15.3% of publications on diseases. This over-representation is also found to a lesser extent for tuberculosis, intestinal nematode infections, arboviroses, tuberculosis, and sexually transmitted diseases (Additional file 2: Table S1).

#### Recommendations

Out of 529 articles on descriptive or implementation research, 256 (48.4%) included some recommendations in the abstract. Of these, 28.9% (*n* = 74) issued actionable messages, either directed to policy managers or to clinicians; 12.1% (*n* = 31) suggested that more studies were needed, and 59.0% (*n* = 151) described the possible implications of the results. Examples of actionable messages are listed in Table 3.

**Table 3:** **Examples of actionable messages found in article abstracts**

**First author, journal publication year**	**Topic**	**“Actionable message”**
Perry, J AM Acad Psychoanal Dyn Psychiatry 2007	Injuries	“*Clinicians should consider the possibility of trauma-related psychological distress in patients who present with undiagnosable physical complaints.*”
MICOL, PLoS One 2010	HIV	“*In a high endemic area of cryptococcosis and HIV infection, serum CRAG screening and prophylaxis are two cost effective strategies to prevent AIDS associated cryptococcosis in patients with CD4+ count ≤100 cells/μl, at a short-term horizon, screening being more cost-effective but less effective than prophylaxis*”
Ir, Health policy, 2010	Health finance	“*While international organisations and donors can take a leading role in innovative interventions in low-income countries, the involvement of government policy makers is necessary for their scaling-up*”
COUTURE Sex TRansm DIs 2011	HIV	“*Amphetamine use was associated with incident Sexually Transmitted Infections. Venue of sex work and drug prevention should be considered in prevention programs*”.
LITTRELL, Malar J, 2011	Malaria	[…] *interventions to improve case management are urgently required, particularly in the private sector. Evidence-based interventions that target provider and consumer behaviour are needed to support uptake of diagnostic testing and treatment with full-course first-line anti-malarials*.”
SASAKI, J Pediatr Nurs, 2010	Nutrition	“[…] *it is important to educate pregnant mothers, probably through exposure to trained midwives and media, so they may recognize the significance of Exclusive Breast Feeding (EBF) and will develop intention and plan to feed their babies, keeping in mind the benefits it may yield. Paternal involvement in breast-feeding classes may increase their awareness and consequently complement EBF practices. Finally, development of conducive working environments and policies for working mothers should be carefully explored because it could have positive influence in better care and promotion of EBF*.”

An actionable message in the article abstract was significantly more frequent when the topic of the publication focused on the maternal and reproductive health (OR 3.08, 95% CI 1.55–6.13, *p* = 0.001), the corresponding author’s affiliated institution being based in Cambodia (OR 1.78, 95% CI 1.06–2.98, *p* = 0.02), and having the full-text article freely accessible via HINARI, PubMed, or Google scholar (OR 3.07, 95% CI 1.08–8.70, *p* = 0.03) (Table 4). Only 1 of the 23 articles published in high impact factor journals included an actionable message in their abstract.

**Table 4:** **Univariate analysis of factors associated with the existence of an actionable and targeted message in the abstract,** ***n*** **= 529 articles (articles on fundamental research were excluded) Cambodia 2000 to 2012**

	**Actionable and targeted message**		**Comparison**		**Total**
	**Yes**	**No**	**OR (95** **%** **CI)**	***p*** **values**	***n*** **= 529 (100.0** **%** **)**
	***n*** **= 73 (13.8** **%** **)**	***n*** **= 456 (86.2** **%** **)**			
Main topic					
Diseases/conditions	43 (12.5)	303 (87.5)	1 (reference)	-	345 (100.0)
Health system	10 (18.9)	43 (81.1)	1.63 (0.76–3.48)	0.20	53 (100.0)
Both	20 (15.3)	111 (84.7)	1.26 (0.71–2.24)	0.42	131 (100.0)
Subtopic					
Communicable diseases	36 (11.4)	281 (88.6)	0.60 (0.36–0.99)	0.048	317 (100.0)
Non-communicable diseases	3 (7.7)	36 (92.3)	0.50 (0.15–1.67)	0.25	39 (100.0)
Maternal and reproductive health	14 (28.6)	35 (71.4)	3.08 (1.55–6.13)	0.001	49 (100.0)
Perinatal conditions	1 (14.3)	6 (85.7)	1.04 (0.12–8.77)	0.97	7 (100.0)
Nutrition	8 (21.0)	30 (78.9)	1.74 (0.76–3.97)	0.18	38 (100.0)
Injuries	5 (11.4)	39 (88.6)	0.78 (0.29–2.06)	0.62	44 (100.0)
Health services delivery	12 (14.3)	72 (85.7)	1.04 (0.53–2.04)	0.88	84 (100.0)
Governance and leadership	12 (19.3)	50 (80.6)	1.59 (0.80–3.16)	0.18	62 (100.0)
Finances and equity	9 (22.5)	31 (77.5)	1.92 (0.87–4.23)	0.10	40 (100.0)
Human resources	3 (9.1)	30 (90.9)	0.60 (0.18–2.04)	0.42	33 (100.0)
Pharmacy and technologies	1 (3.6)	27 (96.4)	0.22 (0.02–1.64)	0.14	28 (100.0)
Information system	3 (18.7)	13 (81.2)	1.46 (0.40–5.25)	0.56	16 (100.0)
Corresponding author’s affiliation					
Cambodian	28 (19.2)	118 (80.8)	1.78 (1.06–2.98)	0.02	146 (100.0)
Cambodian public institutions^a^	12 (18.5)	53 (81.5)	0.91 (0.40–2.11)	0.84	65 (100.0)
Full-text freely accessible^b^	69 (15.1)	387 (84.9)	3.07 (1.08–8.70)	0.03	456 (100.0)

^a^All public hospitals (provincial, national) and governmental institutions, such as the National Center for HIV/AIDS, National Center for Parasitology, National Center for Health Promotion, Faculty of dentistry, and Cambodian Centers for Disease Control.

^b^Via HINARI portal, PubMed central repository, Open Access journal, or Google.

#### Accessibility

Of all 628 publications, 546 (87.0%) were freely accessible from Cambodia using PubMed, HINARI, or Google Scholar. The HINARI portal provided full-text access to 72.0% (*n* = 452) of all articles. Articles available via journal websites accounted for 65.9% (*n* = 414). Full text was available at the PubMed central repository for 55.6% (*n* = 349) of records. Finally, a search on article titles using Google allowed us to access the free full text for 64.8% of all articles (*n* = 407).

## Discussion

This assessment of health research outputs from Cambodia showed that local research has been increasingly productive since 2000, particularly in the field of communicable diseases. Only 7% of all articles addressed NCD. This shows that despite being increasingly recognized as a major burden by Cambodian institutions, NCD remained a subordinate topic of interest for researchers [26,27].

A national survey conducted by the UHS and only published on a WHO website revealed that 82.4% of the Cambodian population presented one to two risk factors for developing NCD [26]. This under-representation of publications on NCD is quite common in LMICs. Indian research, for instance, has shown that in 2002, cardiovascular diseases were addressed by 6.1% of all original publications while contributing to 11.4% of the Indian burden of disease (IBD) [24]. However, the Indian research found that other chronic diseases were well researched [24], which was not the case in Cambodia. This could be suggestive of a lack of interest, prioritization, anticipation, or fund availability for research on this topic in Cambodia. However, it might also reflect the time lag between the buildup of a research project and final publication of the results.

*The* “*HIV/TB/malaria troika*” dominated the publications on CD (42% of all articles on diseases/conditions). Research on these diseases was facilitated by extensive financial support and assistance from Global Fund international experts. These diseases appeared as over-described when compared to the disease burden. This tendency has also been documented at the regional level [26]. In Cambodia, one of the many rationales for extensive studies on malaria is the risk of regional and global anti-malarial resistance spread.

*Other gaps were identified* in the scientific literature from Cambodia. As in India, it is possible that perinatal conditions are under-described [24]. Most articles published on this topic reported observational studies. However, the country also needs implementation research exploring the feasibility of implementation and scaling-up effective interventions. Few publications were about the health system itself (8.4%). A recent situation analysis by the National Institute of Public Health concluded that developing health system research (HSR) in Cambodia was challenging, as the National Health Strategic Plans do not provide clear policy framework for HSR. To date, there is no national strategic plan/agenda for HSR and health research in general, and no specific government budget line for HSR. In 2012, the National Ethics Committee for Health Research approved 205 health research projects. Out of a total budget of USD 28 million, only 15% of these projects potentially dealt with HSR. These projects mainly focus on financial and delivery arrangements for health care and population health. There are no projects focused on leadership/governance. Reasons for an absence of research on leadership/governance, which is the most important health system building block, will be further explored in meetings and workshops.

*Only less than a third of publications were led by an institution based in Cambodia.* If publications from the Pasteur Institute in Cambodia were counted as foreign-led achievements (as the Institute hosts several productive international researchers), the contribution of national scientists would even appear even lower (dropping from 27.5% to 19.4%). This contribution is below the average for a LMIC in the Western Pacific Region (WPR). In WPR, 47.6% of all epidemiological publications were first-authored by someone with an affiliation from a LMIC [26]. It is possible that this situation will improve. Indeed, it has recently become possible to study for a Master’s degree in epidemiology and public health in Cambodia, and some professorial positions now require candidates to publish in international journals. Greater engagement with the peer review process brings credibility and capacity-building opportunities [28-30]. However, there are other barriers that need solving. In particular, there should be national funding allocated to research and better career opportunities and research and language training for local health sciences researchers. International journals could also be more open to publishing research from LMICs [31,32].

*More than 85% of all articles were freely accessible* in full text from Cambodia. HINARI has improved access to international journals for researchers from LMICs [33]. Efforts have been also made by some journals to increase the accessibility of their content. Since 2000, the number of journals providing open access (OA) through the DOAJ has increased by 18% every year and the number of articles has increased by 30% [34]. OA will become even more widespread thanks to new funding rules which often require that researchers deposit their publications in the PubMed Central repository. OA publishing is also a requirement from several research universities [35]. However, there is still room for improving the quality of access to the best level of information. OA remains expensive for scientists when submitting their work, and there is a need to develop new search tools that can retrieve more specific types of publications (e.g., at selected level of evidence, etc.) according to the needs of health academics, policymakers, and managers.

*In 2010*, Cambodia was awarded a Millennium Development Goal (MDG) prize for “Excellence in its AIDS response” [36]. Cambodia is also one of the few countries which has seen dramatic improvements in child health in a very short time [37]. Although very little has been published on maternal and child health, Cambodia is one of the 16 countries likely to reach the MDG 5 on maternal health [37]. In many ways, this mismatch highlights the fact that publications are only one of many means of translating research findings into public health practice [38]. International experts exert a strong influence on Cambodian policymaking. Conferences, face-to-face communications, research reports, and policy briefs are other important ways in which research outputs can have an impact [39,40]. They also complement with international agencies’ guidelines and non-peer-reviewed sources of information such as reports of national surveys, NGOs, and international agencies or surveillance data [41].

*Our findings must be read in light of their limitations*. Firstly, the analysis only covered scientific publications in peer-reviewed journals and therefore missed all other types of evidence produced by research projects. We also based the analysis on data collected only from the abstracts. We considered that scientific abstracts are far more likely to be read than any other section of any article. We did not assess whether policy makers read scientific publications. However, if they do, it is very likely that they first read the abstracts [42]. We also considered that although the format of abstract is usually imposed by journals, they always contain the key messages in the article [43].

Secondly, we defined the origin of the research as the country of affiliation of the first author, which does not always reflect the reality of who led the research leading and true authorship.

Thirdly, we referred to the 2004 GBDS to estimate the disease burden in Cambodia [19]. This was the most comprehensive recent and reliable source available. We acknowledge that the burden of disease does not capture the full magnitude of a health problem and that it has changed over the past 10 years. As with large number of publications on malaria in Cambodia, we understand that a particular disease can have such a large global impact that it merits a level of research that exceeds its proportionate burden within the country in question.

Our review included articles published until October 2012 only. An additional search covering the period November 2012–December 2014 (following the same search strategy) identified 179 new articles. However, a brief analysis of the main topic of publication showed that 86% of these new articles cover diseases and health conditions, which is even higher than what we found for 2000–2012. Similarly, the “troika” HIV/TB/malaria still represents 44% of all articles on diseases, and studies on NCD are even rarer for the more recent period (4% of articles on diseases). This may reflect a time lag to publication.

## Conclusion

The body of knowledge produced by the research in Cambodia does not fully match the public health priorities. Despite an important increase, Cambodian health research is mostly descriptive and tends to be focused on CD and driven by funding. As such, it remains partial, and there is a lack of implementation research. A governmental coordination body should be given the means to develop a health research agenda that is aligned with public health priorities. In the meantime, gaps could be bridged through workshops and meetings to improve coordination between stakeholders and policymakers. NCDs and interventional studies are areas with particularly strong needs for research. However, as in other LMICs, limited financial and human resources remain significant challenges. Research involving international scientists should be considered as an opportunity to train and cooperate with local researchers.

To increase the chance of knowledge uptake in public health policies, we recommend the following: 1) National health authorities should create an online repository where researchers can upload their research findings (and abstracts in the local language); 2) Academics should emphasize the importance of research in university medical training; 3) Upon submission to a journal, researchers should systematically provide a translation of their abstracts in the language of the country where the research was conducted (an abstract in Khmer of this article is available upon request to the corresponding author).

Publishing is only a part of the KT process. The bridge between research and public health needs to be paved with more than publications and good intentions. Building this bridge should start from both sides of the gap: it should occur early on in the research and policymaking processes, and it should involve a variety of complementary KT interventions.

## Additional files

Additional file 1:
**Search strategy.** This document presents the key words and filters used for each source of articles explored, as well as the number of hits obtained. Additional file 2:
**Systematic review PRISMA check list.** The PRISMA check list presents the characteristics of this systematic review. Additional file 3:
**Comparison of publications on health with the 2004 burden of diseases estimation, Cambodia, 2000 to 2012.** This table displays the differential between the number of publications from 2000 to 2012 and the 2004 contribution to the burden of disease for each topic.
