# Supplementary material for: Gaps between research and public health priorities in low income countries: evidence from a systematic literature review focused on Cambodia
Source: Implement Sci. 2015 Mar 11;10:32. doi: 10.1186/s13012-015-0217-1 (PMC4357145; doi:10.1186/s13012-015-0217-1)
Supplement: Additional file 1: — Search strategy. This document presents the key words and filters used for each source of articles explored, as well as the number of hits obtained. [file 13012_2015_217_MOESM1_ESM.docx]

Search strategy

**PubMed Search**

The Medline ([www.nlm.nih.gov/services/usemedline.html](http://www.nlm.nih.gov/services/usemedline.html)) is the US National Library of Medicine database of over 19 million references to articles published in more than 5000 current biomedical journals of more than 80 countries.

PubMed (which includes MEDLINE) of the US National Library of Medicine, the most widely used online health literature search database in the world, and websites of international agencies, (and other sources), were searched to ascertain the health research output from Cambodia since the year 2000.

Search: (((((Cambodia[Title/Abstract]) OR Cambodian[Title/Abstract]) OR Cambodia[MeSH Terms]) AND english[Language])) AND ("2000"[Date - Publication] : "3000"[Date - Publication]) Filters: Abstract available, Humans *Pubmed search****>> 1056 hits***

1. **Directory of Open Access Journal (DOAJ) search**

The DOAJ (<http://www.doaj.org/>) covers the open access scientific and scholarly journals that “use a quality control system to guarantee the content” and “use a funding model that does not charge readers or their institutions for access”.

*(Cambodia and health in all search fields* ***>> 162 hits*** *)*

1. **French medical scientific journals search using**

<http://www.gfmer.ch/Medical_journals/Journaux_medicaux_acces_libre.htm>

| **Name of the journal** | **access** | **strategy** | **n. of hits** |
| --- | --- | --- | --- |
| Bulletin de la société de pathologie exotique | <http://www.pathexo.fr/bull_sommaire.php>  (January 2013) | Cambodge as a key word | **8** |
| Médecine tropicale | <http://www.revuemedecinetropicale.com/>  (January 2013) | Cambodge as a key word | **94** |
| Santé tropicale | <http://www.santetropicale.com/>  (January 2013) | Cambodge as a key word | **0** |
