# Supplementary material for: Gaps between research and public health priorities in low income countries: evidence from a systematic literature review focused on Cambodia
Source: Implement Sci. 2015 Mar 11;10:32. doi: 10.1186/s13012-015-0217-1 (PMC4357145; doi:10.1186/s13012-015-0217-1)
Supplement: Additional file 3: — Comparison of publications on health with the 2004 burden of diseases estimation, Cambodia, 2000 to 2012. This table displays the differential between the number of publications from 2000 to 2012 and the 2004 contribution to the burden of disease for each topic. [file 13012_2015_217_MOESM3_ESM.docx]

**Additional file 3: Comparison of publications on health with the 2004 Burden of diseases estimation, Cambodia, 2000 to 2012**

|  |  |  | **Publications 2000 - 2012** | **Burden of diseases 2004** | **Differential** between publications and burden of disease |
| --- | --- | --- | --- | --- | --- |
| **Topics** | | **n** | % | % Estimated DALYs / 100,000 pop |  |
| **All diseases and conditions** | | **575** | **100.0** | **100.0** |  |
| **Communicable diseases** | | **410** | **71.3** | **58.0** | 13.3 |
|  | HIV/AIDS | 138 | 24.0 | 5.4 | 18.6 |
|  | Malaria | 88 | 15.3 | 0.4 | 14.9 |
|  | Tuberculosis | 43 | 7.5 | 4.7 | 2.8 |
|  | Intestinal nematode infection | 41 | 7.1 | 1.0 | 6.1 |
|  | Influenza diseases | 32 | 5.6 | na | na |
|  | Arboviroses (dengue, japanese encephalitis) | 24 | 4.2 | 0.2 | 4.0 |
|  | Sexually transmitted diseases (other than HIV) | 19 | 3.3 | 0.5 | 2.8 |
|  | Childhood cluster diseases^1^ | 10 | 1.7 | 0.7 | 1.0 |
|  | Hepatitis B and C | 10 | 1.7 | 0.6 | 1.1 |
|  | Respiratory infections (inc. influenza) | 6 | 1.0 | 12.8 | -11.8 |
|  | Diarrhoea | 6 | 1.0 | 7.8 | -6.8 |
|  | Meningitis | 2 | 0.3 | 1.0 | -0.7 |
| **Non communicable diseases** | | **44** | **7.7** | **34.5** | -26.8 |
|  | Congenital diseases | 13 | 2.3 | 1.7 | 0.6 |
|  | Neoplasma | 11 | 1.9 | 3.4 | -1.5 |
|  | Diabetes | 5 | 0.9 | 0.8 | 0.1 |
|  | Cardiovascular diseases | 3 | 0.5 | 7.2 | -6.7 |
| **Maternal conditions** | | **49** | **8.5** | **3.7** | 4.8 |
|  | Family-planning, birth control | 23 | 4.0 | na | na |
|  | Pregnancy related | 10 | 1.7 | na | na |
|  | Labor related | 6 | 1.0 | na | na |
|  | Abortion | 5 | 0.9 | na | na |
|  | Post-natal related | 4 | 0.7 | na | na |
|  | Hemorrhage | 2 | 0.3 | na | na |
|  | High blood pressure and pre-eclampsia | 3 | 0.5 | na | na |
| **Perinatal conditions** | | **7** | **1.2** | **12.5** | -11.3 |
|  | Prematurity and low birth | 0 | 0.0 | 3.2 | -3.2 |
|  | Birth asphyxia and birth trauma | 1 | 0.2 | 4.5 | -4.3 |
|  | Neonatal infections and other conditions | 0 | 0.0 | 4.8 | -4.8 |
| **Nutritional deficiencies** | | **39** | **6.8** | **4.3** | 2.5 |
|  | Protein-enery malnutrition | 2 | 0.3 | 1.9 | -1.6 |
|  | Iodine deficiency | 1 | 0.2 | 0.3 | -0.1 |
|  | Vitamine A deficiency | 11 | 1.9 | 0.0 | 1.9 |
|  | Iron deficiency anaemia | 15 | 2.6 | 1.7 | 0.9 |
| **Injuries and traumas** | | **44** | **7.7** | **7.5** | 0.2 |
|  | Unintentional injuries | 30 | 5.2 | 5.4 | -0.2 |
|  | Intentional injuries | 4 | 0.7 | 2.2 | -1.5 |

Notes:

1: Childhood diseases are pertussis, poliomyelitis diphteria, measles, tetanus

2: na means non available in the Global burden of diseases estimations as such
